# Elevated Initial Serum Phosphate Levels Predict Higher Mortality and Impaired Neurological Outcome in Cardiac Arrest Patients with Return of Spontaneous Circulation

**DOI:** 10.3390/diagnostics13030479

**Published:** 2023-01-28

**Authors:** Dragos Andrei Duse, Michael Gröne, Nicolas Kramser, Matthias Ortkemper, Christine Quast, Fabian Voß, Nadia Heramvand, Karel Kostev, Malte Kelm, Patrick Horn, Christian Jung, Ralf Erkens

**Affiliations:** 1 Department of Cardiology, Pulmonology and Angiology, Medical Faculty, Heinrich Heine University of Düsseldorf, 40225 Düsseldorf, Germany; 2 University Clinic of Marburg, Philipps-University Marburg, 35039 Marburg, Germany; 3 CARID (Cardiovascular Research Institute Düsseldorf), 40225 Düsseldorf, Germany

**Keywords:** prognosis, resuscitation, phosphate, outcome

## Abstract

**Purpose:** Although a moderate proportion of cardiac arrest (CA) patients achieve a return of spontaneous circulation (ROSC), few survive to discharge, mostly with poor neurological development. As serum phosphate levels were described as elevated after cardiopulmonary resuscitation (CPR), we asked whether these elevations would predict a higher risk of mortality and impaired neurological outcome in CA patients following ROSC. **Methods:** Initial serum phosphate levels, survival, and neurologic status at discharge of 488 non-traumatic CA patients treated at a single German hospital after achieving ROSC were analyzed. The cut-off value of phosphate for mortality prediction was determined using the receiver operator characteristic (ROC) curve, and patients were divided accordingly for comparison. Results were validated by analyzing phosphate levels in a multi-centric cohort containing 3299 CA patients from the eICU database of the United States. **Results:** In the German cohort, ROC analysis showed a 90% specificity for phosphate levels >2.7 mmol/L to predict mortality (AUC: 0.76, *p* < 0.0001), and phosphate level elevations were associated with higher in-hospital mortality (crude odds ratio 3.04, 95% CI 2.32 to 4.08). Patients with initial phosphate levels >2.7 mmol/L had significantly higher mortality in both analyzed collectives (*p* < 0.0001). Similarly, patients from the German cohort who initially had higher phosphate levels also showed a higher proportion of impaired neurological status at discharge and morphological signs of brain injury. **Conclusions:** In CA patients following ROSC, initial serum phosphate levels >2.7 mmol/L predict higher mortality and impaired neurological outcome. Our data suggests that phosphate determination might improve the preciseness of the overall and neurologic prognostication in patients after CPR following ROSC.

## 1. Introduction

CA is an acute life-threatening condition recoverable only by timely CPR. Following CPR, about one-third of out-of-hospital CA (OHCA) and about 40–50% of in-hospital CA (IHCA) patients achieve initial ROSC [1,2,3]. Despite these moderate ROSC rates, only about 4–8% of the initial OHCA [1,4] patients and about 15–20% of the initial IHCA [5] patients survive. The difference between the tremendous mortality rate in view of multiple higher ROSC rates suggests a compelling need to improve initial prognostications markers for the further clinical development of the patients.

Inorganic phosphate, commonly referred to as phosphate in the medical field, is a chemical element with structural importance for bones and teeth and which plays an essential role in cellular signaling [6]. Phosphate enters the human body via absorption from food, and it leaves the body via renal excretion, with major interactions between these organ systems in its regulation [7,8]. Similar to lactate, serum phosphate is a feasible laboratory parameter and part of the clinical routine testing post-CPR. 

Several studies have associated changes in serum phosphate levels with cardiovascular events in patients with coronary artery disease and heart failure [9,10,11], suggesting a cardiac and circulatory relationship with phosphate metabolism. In line with these associations, basic research studies found increased serum phosphate in canines with CA after CPR [12], which was also corroborated in patients suffering from CA [13]. It appears that changes in phosphate homeostasis could reflect the impaired metabolic functions in the human body. In an investigation of CA patients following CPR, initial serum phosphate levels had a modest predictive power on the neurological outcome [14]. Several recent studies described increasingly higher phosphate levels at the time of index in patients with poorer neurological outcomes. Phosphate proved to be a suitable parameter for inclusion in complex multivariate analyses to improve predictive accuracy [15,16]. 

Although some studies noted increased phosphate levels after CPR and the inclusion of phosphate into complex multimodal analyses [15,16] underlined its value, little is known about the independent predictive value of phosphate on mortality and neurologic outcome in CA patients who achieve ROSC. Using data from the University Clinic of Duesseldorf, we retrospectively investigated the predictive role of the initial phosphate levels on survival and neurological outcome in patients with ROSC after CA. We then evaluated and validated these results from a single center in a database containing CA patients from multiple ICU in the United States.

## 2. Methods

### 2.1. Study Design and Populations

We retrospectively screened 1086 patients suffering from non-traumatic CA who received CPR and were treated at the University Clinic Düsseldorf between the 1 January 2013 and the 31 December 2017. All patients had a documented endpoint regarding survival and were aged ≥18 years. The ethics committee of Heinrich-Heine University approved the study (2018–109-RetroDEuA).

The study’s main objective was to investigate the predictive power of the initial phosphate levels of patients with CA and ROSC on two major endpoints: survival and neurological development at discharge. Data completion of these parameters was mandatory for patients’ inclusion. All included patients from the German cohort achieved ROSC post-CPR.

Secondary objectives were the peak neuron-specific enolase (NSE) levels of patients divided by the cut-off value and the initial phosphate levels of CA patients with morphological signs of brain injury, as measured by cranial MRI and CT. NSE levels are accepted neurological outcome parameters in CA patients [17]. Moreover, CPR-related neurological complications (such as brain ischemia), which can be visualized by cranial imaging [18], count among the main reasons for cognitive impairment. Cranial imaging was evaluated by radiologists at the University Clinic of Duesseldorf. We interpreted cerebral edema (reversible/irreversible) and morphological patterns of hypoxic brain injury as CPR-related complications displayed by cranial imaging. Since these analyses were secondary to our main objective, data completion of these parameters was not mandatory. Finally, we analyzed the relationship between initial phosphate levels and initial lactate levels, as lactate levels have already been validated as a prognosis marker [19,20]. Similar to our second objective, the completion of these data was not mandatory for analysis.

Based on the single-center collective treated in the University Clinic of Duesseldorf, we generated our initial findings regarding phosphate as a predictive marker. We evaluated whether these preliminary results would apply in a multi-center database of 3299 patients with CA from the eICU Collaborative Research Database. As previously described [20], this database contains the hospitalization details of over 200,000 admissions from over 300 intensive care units (ICU) in the United States of America in 2014 and 2015. This database is heterogenous with a from our first database completely independent patient population. Due to the lack of information on ROSC status, we performed survival analyses in all patients from this database.

### 2.2. Parameters Analyzed

As part of a standardized (post-)CPR algorithm at the University Clinic of Duesseldorf, blood samples were collected from OHCA patients within the first ten minutes of hospital admission and from IHCA patients within the first ten minutes of CPR. The samples were then sent directly to the central hospital laboratory. The estimated glomerular filtration rate (eGFR) was calculated by the central hospital laboratory calculated following the Chronic Kidney Disease Epidemiology Collaboration (CKD-EPI) equation [21]. Cranial imaging results, several laboratory parameters at the time of index and in the following five days, days of hospitalization after ROSC, and cognitive status at discharge were collected from medical records in the hospital’s clinical data information system (*Medico* (Cerner GmBh Deutschland) and *PEGASOS* (Nexus/Marabu GmBh)). Neurological outcomes were quantified by the cerebral performance category (CPC), as previously described [22]. CPC scores 1 and 2 represented good and moderate cerebral performance, respectively. A CPC score of 3 indicated severe neurological impairment for requiring assistance, whereas a score of 4 indicated an unconscious state. Patients with a CPC score of 5 were confirmed (brain-) dead. A poor neurological outcome at discharge was defined by a CPC score ≥3.

Specific baseline characteristics, demographic data, and various laboratory parameters, including initial serum phosphate levels on the first day post-CPR, were collected from the multicenter eICU collective and analyzed accordingly. We used the maximum value for analysis for patients with multiple documented phosphate or lactate levels on the first day post-CPR.

### 2.3. Statistical Analysis

Continuous data points are expressed as mean ± standard error of the mean (SEM). Normal distribution was assessed using the D’Agostino–Pearson test. Differences between independent groups were calculated using unpaired t-tests for normally distributed continuous variables and the Mann–Whitney U test for non-normal distributed data. The relationship between the initial lactate and phosphate values is reflected by the Pearson correlation coefficient. Phosphate’s prognostic value on mortality was assessed by a ROC curve analysis with an area under the curve (AUC) of serum phosphate levels after CPR to predict in-hospital survival. The phosphate level that could predict mortality with a specificity of 90% was set as the cut-off value, and patients were divided into two groups accordingly. Survival was assessed by Kaplan–Meier analyses, and the differences were analyzed using the Log-rank (Mantel–Cox) test. The relationship between initial phosphate level elevations and in-hospital mortality was presented by OR. It was calculated as a raw value by simple logistic regression and adjusted for initial creatinine levels by multiple logistic regression analysis. All tests were two-sided, and a *p*-value of <0.05 was considered statistically significant. All statistical analyses were performed in GraphPad Prism for Windows (GraphPad Software, San Diego, CA, USA).

## 3. Results

### 3.1. Overall Characteristics of CA Patients Treated at the University Clinic of Duesseldorf

**Figure 1:**
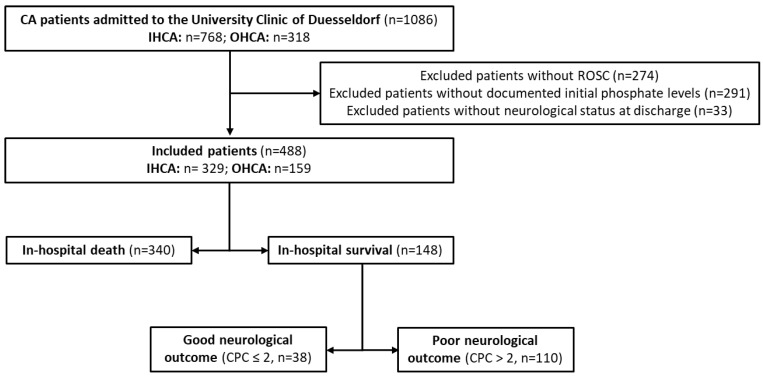
Flow chart of the CA patient’s population in our analysis.

We screened 1086 patients, 768 patients with IHCA (71%) and 318 patients with OHCA (29%). Of these, 274 patients did not achieve ROSC, 291 patients had no documented initial phosphate levels, and 33 patients had no information on neurological status at discharge and were excluded. The remaining 488 patients were included in the analysis (Figure 1).

The proportion of IHCA and OHCA patients included was similar to that of the screened patients (IHCA *n* = 329, 67%; OHCA *n* = 159, 33%). Overall, approximately two-thirds of the patients from the analyzed collective died during hospitalization (*n* = 340, 70%), whereas the other third survived to discharge (*n* = 148, 30%). Out of the survivors, 26% of the patients (38/148) showed good neurological development at discharge (CPC ≤ 2), and the other 74% of the patients showed a bad cognitive status (CPC ≥ 3). Demographic data showed a higher percentage of men (*n* = 304, 62.3%) and IHCA (*n* = 329; 67.4%). The average age was 69.88 years (SD: 12.99 years). Causally, cardiac diseases were responsible for most CA. Table 1 shows the baseline characteristics of patients treated at the University Clinic of Duesseldorf.

### 3.2. Initial Phosphate Elevations over 2.7 mmol/L Predict a Higher Risk of Mortality

**Figure 2:**
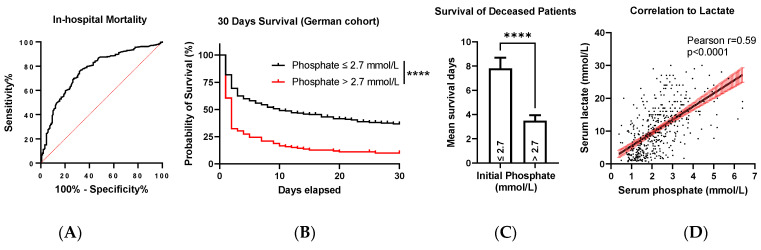
(**A**) ROC curve of serum phosphate levels to predict mortality AUC: 0.7610 (95%-CI: 0.7188–0.8032, *p* < 0.0001). Cut-off value to predict mortality with 90% specificity: 2.7 mmol/L. (**B**) Kaplan–Meier curve representing the survival of CA patients achieving ROSC in dependency of their initial serum phosphate >2.7 mmol/L (red) or ≤2.7 mmol/L (black) (Log-rank (Mantel–Cox) test: **** *p* < 0.0001). (**C**) Mean survival days of patients with ROSC who ultimately died during the hospital stay dependent on initial phosphate values (median: 2 vs. 3 days; Mann–Whitney test: **** *p* < 0.0001). (**D**) Correlation between the initial serum lactate and phosphate levels, Pearson r = 0.59 (95% CI: 0.53 to 0.65, R^2^ = 0.35, *p* < 0.0001).

Our first analysis concerned the relationship between initial phosphate levels after ROSC and survival at discharge. The ROC curve, which examined the predictive power of initial phosphate levels for in-hospital mortality, showed an AUC of 0.76 (95% CI: 0.72 to 0.80, Figure 2A, *p* < 0.0001). The crude OR of phosphate elevations on in-hospital mortality was 3.04 (95% CI: 2.32 to 4.08, Hosmer–Lemeshow *p* = 0.0889). Because phosphate is eliminated renally and elevations might occur in kidney diseases [23], we repeated our analysis by including the initial creatinine level in a multivariate logistic regression analysis. Interestingly, even after correction for creatinine, phosphate had an aOR of 2.87 (95%-CI: 2.18 to 3.864), with a low calibration (Hosmer–Lemeshow *p* = 0.0748).

According to the ROC analyses, phosphate values >2.7 mmol/L predicted in-hospital mortality with a specificity of 90% in patients with ROSC. We set this value as a cut-off and divided the patient population into two cohorts accordingly. Patients with initial phosphate values >2.7 mmol/L had higher mortality in the first 30 days after CPR (Figure 2B, *p* < 0.0001). This finding was reinforced by the average survival days of the patients who eventually died in the hospital: patients with initial phosphate values ≤2.7 mmol/L survived significantly longer on average than patients with initially higher phosphate levels (Mann–Whitney test: *p* < 0.0001, Figure 2C). Correlation analysis of initial serum phosphate and lactate levels revealed a moderate relationship (Pearson r = 0.59, 95% CI: 0.53 to 0.65, *p* < 0.0001, Figure 2D). 

### 3.3. Higher Phosphate Levels at the Index Forecast a Poor Neurological Development

**Figure 3:**
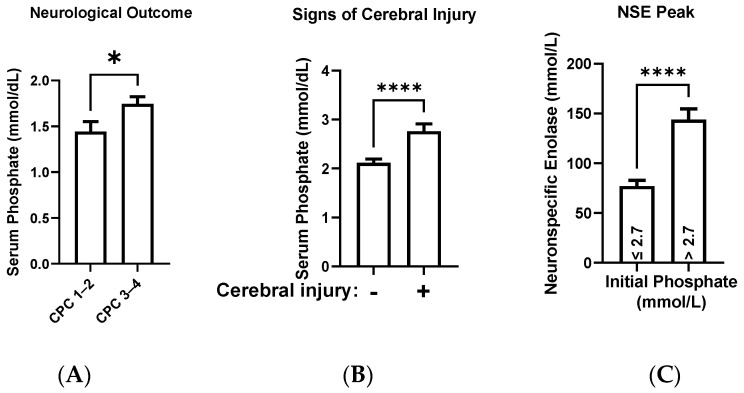
(**A**) Comparison of the average serum phosphate levels dependent on the neurological outcome measured by the cerebral performance index (mean phosphate: 1.4 ± 0.11 (SEM) in CPC 1–2 vs. 1.7 ± 0.08 mmol/L in CPC 3–4, Mann–Whitney test: * *p* = 0.0143). (**B**) Comparison of the average serum phosphate levels in patients without and with morphological signs of brain injury, as confirmed by cranial imaging post-CPR (mean phosphate: 2.1 ± 0.08 (SEM) vs. 2.8 ± 0.15 mmol/L, Mann–Whitney test: **** *p* < 0.0001). (**C**) NSE-Peak values in the first five days after CPR of patients with initial phosphate levels >2.7 mmol/L and ≤2.7 mmol/L (mean NSE Peak: 77.1 ± 5.8 (SEM) in phosphate ≤2.7 mmol/L vs. 143.8 ± 10.1 mmol/L in phosphate >2.7 mmol/L, Mann–Whitney test: **** *p* < 0.0001).

Patients with a CPC score ≤2 initially had significantly lower phosphate levels than patients with a CPC score ≥3 (mean phosphate: 1.4 ± 0.11 (SEM) vs. 2.3 ± 0.05 mmol/L Mann–Whitney Test, *p* < 0.0001). As the patient collective with a poor neurological outcome also included patients who died during the hospital stay, we corrected our analysis to death by excluding patients with a CPC score of 5. In the subgroup of patients surviving to discharge, those with an impaired neurological outcome (CPC score ≥ 3) had significantly higher initial serum phosphate values (mean phosphate: 1.4 ± 0.11 (SEM) in CPC ≤ 2 vs. 1.7 ± 0.08 mmol/L in CPC 3–4, Mann–Whitney test: *p* = 0.0143, Figure 3A). 

Our secondary endpoint showed similar results. In the subgroup of patients who received initial cranial imaging (213/488, 43%), patients with morphological signs of brain injury had higher phosphate levels (mean phosphate: 2.1 ± 0.08 (SEM) vs. 2.8 ± 0.15 mmol/L, Mann–Whitney test: *p* < 0.0001, Figure 3B). Additionally, patients with initial phosphate >2.7 mmol/L had significantly higher NSE peak levels within the first five days post-CPR (mean NSE Peak: 77.1 ± 5.8 (SEM) in phosphate ≤2.7 mmol/L vs. 143.8 ± 10.1 mmol/L in phosphate >2.7 mmol/L, Mann–Whitney test: *p* < 0.0001, Figure 3C).

### 3.4. Stratification of the CA Forms IHCA and OHCA 

**Figure 4:**
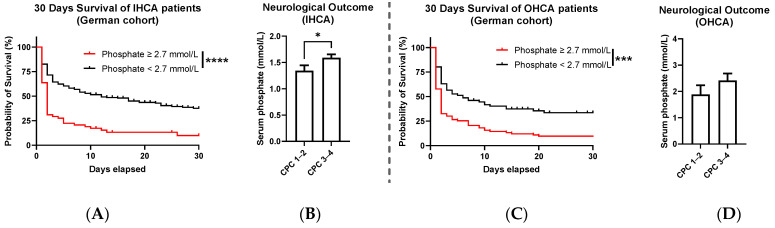
(**A**) Kaplan–Meier curve representing the survival of IHCA patients achieving ROSC in dependency of their initial serum phosphate >2.7 mmol/L (red) or ≤2.7 mmol/L (black) (Log-rank (Mantel–Cox) test: **** *p* < 0.0001). (**B**) Comparison of the average serum phosphate levels of IHCA patients dependent on the neurological outcome measured by the cerebral performance index (mean phosphate: 1.3 ± 0.1 (SEM) in CPC 1–2 vs. 1.6 ± 0.07 mmol/L in CPC 3–4, Mann–Whitney test: * *p* = 0.0168). (**C**) Kaplan–Meier curve representing the survival of OHCA patients achieving ROSC in dependency of their initial serum phosphate >2.7 mmol/L (red) or ≤2.7 mmol/L (black) (Log-rank (Mantel–Cox) test: *** *p* = 0.0001). (**D**) Comparison of the average serum phosphate levels of OHCA patients dependent on the neurological outcome measured by the cerebral performance index (mean phosphate: 1.9 ± 0.3 (SEM) in CPC 1–2 vs. 2.4 ± 0.3 mmol/L in CPC 3–4, Mann–Whitney test: ns *p* = 0.1859).

Because IHCA patients comprised two-thirds of the entire CA collective, we asked whether initial phosphate levels would similarly predict survival and neurologic outcome in both CA forms. ROC analyses performed for IHCA showed an AUC of 0.75 (95% CI 0.7–0.8, *p* < 0.0001). In the same collective, the Kaplan–Meier Survival analysis revealed a huge difference in the chance of survival (Figure 4A, Log-rank (Mantel–Cox) test: *p* < 0.0001). When analyzing the initial phosphate levels of IHCA patients who survived to hospital discharge, those with good and moderate neurological status had lower initial phosphate levels on average (mean phosphate: 1.3 ± 0.1 (SEM) in CPC 1–2 vs. 1.6 ± 0.07 mmol/L in CPC 3–4, Figure 4B, Mann–Whitney test: *p* = 0.0168).

The analysis of OHCA patients provided comparable results. With an AUC in the ROC analysis of 0.7 (95% CI 0.58–0.82, *p* = 0.0009), phosphate proved a suitable prognosis parameter of the chance of survival. The 30-day survival rate was significantly lower in patients with initial phosphate >2.7 mmol/L (Figure 4C, Log-rank (Mantel–Cox) test: *p* = 0.0001). Among surviving OHCA patients, phosphate levels were slightly lower in those with a CPC ≤ 2 (Figure 4D, mean phosphate: 1.9 ± 0.3 (SEM) in CPC 1–2 vs. 2.4 ± 0.3 mmol/L in CPC 3–4, Mann–Whitney test: *p* = 0.1859), a trend which was not significant, which may be explained by the small number of patients (*n* = 28) in this collective.

### 3.5. Phosphates Predictive Value in an Additional Multicenter Collective

We examined whether the cut-off value for phosphate to predict a higher risk of mortality would apply to other collectives because we had obtained our initial findings in a single-center cohort. Therefore, we transposed the cut-off value of phosphate emerged from our preliminary results to predict mortality in the collective of 3299 patients from the eICU research database, which had documented initial phosphate levels. In the eICU database, approximately one-third of patients died during hospitalization after CPR. Additional demographic data on the collective are shown in Table 2.

**Figure 5:**
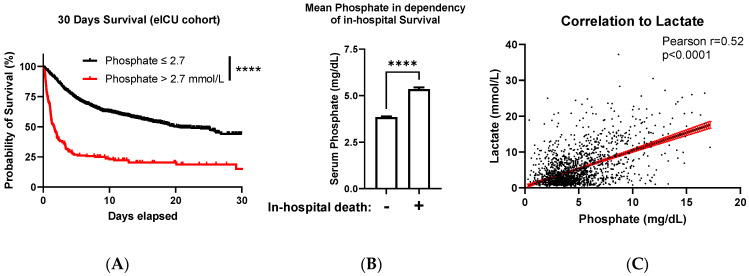
(**A**) Kaplan–Meier curve of patients after ROSC with initial serum phosphate >2.7 mmol/L (red) or ≤2.7 mmol/L (black) from the eICU database (Log-rank (Mantel–Cox) test: **** *p* < 0.0001). (**B**) Mean initial phosphate levels (maximum value of first hospital day) of patients from the eICU database, in dependency of their hospital survival (mean phosphate: 3.8 ± 0.03 (SEM) vs. 5.4 ± 0.09 mg/dL; Mann–Whitney test: **** *p* < 0.0001). (**C**) Correlation between the initial serum lactate and phosphate levels, Pearson r = 0.52 (95% CI: 0.49 to 0.56, R2 = 0.27, *p* < 0.0001).

ROC analysis in the eICU collective yielded an AUC of 0.66 (95%-CI: 0.64 to 0.68, *p* < 0.0001) for elevated initial phosphate levels to predict mortality. Because this database served as a validation tool for our initial finding in the single-center cohort (of patients treated at the University Clinic of Düsseldorf), we divided patients from the eICU database into two groups based on phosphates cut-off value of 2.7 mmol/L and analyzed the differences in early mortality. KMC showed an increase in 30-day mortality for patients with initial phosphate levels >2.7 mmol/L (Figure 5A, *p* < 0.0001). In addition, patients who died during the hospitalization had higher initial phosphate levels (phosphate on the first day: 3.8 ± 0.03 (SEM) in surviving vs. 5.4 ± 0.09 mg/dL in deceased patients, Mann–Whitney test: *p* < 0.0001, Figure 5B). In line with the first analyzed collective, correlation analysis of initial serum phosphate and lactate levels showed a moderate relationship (Pearson r = 0.52, 95% CI: 0.49 to 0.56, *p* < 0.0001, Figure 5C).

## 4. Discussion

By retrospective investigation of a large collective of CA patients with consecutive ROSC treated at the University Clinic of Duesseldorf, this study presents two major findings:

1. Initial phosphate levels >2.7 mmol/L predict a lower chance of survival;
2. Higher initial serum phosphate levels forecast a poor neurological outcome.

By transposing the cut-off value of phosphate to predict mortality to a second, independent collective from multiple ICUs in the USA, we validated the independent prognostication value of initial phosphate levels on the risk of mortality during the hospitalization post-CPR. To the best of our knowledge, this is the first study to address the independent and time-lagged predictive relevance of phosphate for CA patients achieving ROSC for multiple outcomes (risk of mortality and neurological impairment) post-CPR. 

Our study first addressed the question of whether initial phosphate elevations would indicate a higher risk of mortality during the intensive care treatment post-CPR. Although the initial ROC, shown in Figure 2A, had acceptable discrimination [24] (reflected in the AUC), we saw a large difference between the collectives when patients were divided based on the cut-off value of 2.7 mmol/L, as shown in Figure 2B. In quantitative terms, patients with initial serum phosphate >2.7 mmol/L had tremendously higher mortality during hospitalization. Moreover, of the patients who died during the post-CPR treatment, those with an initial phosphate >2.7 mmol/L lived, on average, fever days (Figure 2C). Finally, logistic regression analyses underscored the predictive power of phosphate. As phosphate is a metabolite excreted by the kidneys [8], renal disease, which itself can be triggered by the CA and CPR [25], can lead to an increase in phosphate. Therefore, we sought to adjust the predictive power for renal diseases. After including creatinine in addition to phosphate in a multiple logistic analysis, we found an aOR of phosphate of 2.87 (95% CI: 2.18 to 3.86). Altogether, these results demonstrated the predictive effect of initial phosphate levels on survival. 

In the group of patients treated at the University Clinic of Duesseldorf, initial phosphate values >2.7 mmol/L predicted a higher risk of in-hospital mortality. When we applied this cut-off value to the multicenter collective from the United States, a similar representation of outcomes for CA patients dependent on their initial phosphate levels was found: patients with initially higher phosphate levels after CPR showed higher mortality during intensive care post-CPR (Figure 5A). This analysis validated our results from a single-center patient group in a heterogenous multicenter collective. However, it should be noted that there was no information on ROSC status in the second collective. With this analysis and for patients from this database, we established the predictive role of phosphate for all CA patients receiving CPR. 

Given these findings, the subject of phosphate elevation to predict mortality is not new. Several studies have demonstrated this association between phosphate elevations and a higher risk of mortality in patients with septic shock with and without mechanical ventilation [26,27] and in patients with blunt trauma [28]. It seems that phosphate could act as a moderate to good prognostic marker for mortality in critically ill patients, as it has already been described for patients in medical ICUs [29] and elderly patients [30]. More recently, a study on polytrauma patients showed a relationship between hyperphosphatemia and injury severity with a consecutive higher risk of mortality [31]. The results obtained in our study allowed us to add to underlying diseases with phosphate elevations, where phosphate predicts mortality in CA patients achieving ROSC. 

The results of this study reflect an existing relationship between the initial phosphate and cognitive status at discharge in CA patients with ROSC. This relationship was supported by several of our findings: First, among patients who survived to discharge, we saw that those with poor neurological development had initially higher serum phosphate levels (Figure 3A). Second, the group of patients with morphologic signs of brain injury had higher phosphate levels than those without any pathological results in cranial imaging (Figure 3B). Third, we found significantly higher NSE levels in the first five days of the intensive care treatment in patients with initial phosphate levels >2.7 mmol/L (Figure 3C). Overall, these results highlight the predictive role of phosphate on the neurological outcomes of CA patients with ROSC.

Several studies have analyzed models for CA patients whose initial phosphate values were included to predict worse neurological outcomes [14,15]. One of these studies investigated the predictive power of phosphate alone on neurological outcomes in CA patients following ROSC, and the authors showed an association between the analyzed parameter and delayed neurological impairment in a Korean multicenter cohort [14]. Similarly, we found an association between elevated phosphate and worsened cognitive outcomes by direct and indirect analyses in the cohort of patients treated in Duesseldorf, which, in summary, supports the likelihood of a direct association. Undoubtedly, combining five or more initial parameters for outcome prediction would lead to higher precision, as several studies have shown [15,16]. The presented results could partially explain why the inclusion of phosphate into the multivariate analysis would positively affect the predictive power. However, because phosphate is a feasible and cost-effective parameter, prospective clinical trials should include an initial determination of phosphate levels and investigate their effect on cognitive development. 

Phosphate’s independent predictive value was partially underlined by a moderate correlation with lactate in both analyzed collectives (Figure 2D and Figure 5C). Lactate, mainly produced by skeletal muscles of the human body, accumulates in the serum of cardiac arrest patients due to multiple alterations. (Tissue) Hypoxia induces dysfunctional mitochondrial oxidation, the coexisting acidemia (due to released protons in lactate generation) impairs the hepatic elimination of lactate, which influences aerobic and anaerobic glycolysis in CA patients [32]. Similarly, hyperphosphatemia occurs in CA patients due to organ ischemia and malperfusion, with consecutive cellular shift [31,33] contributing to CA-induced acidosis [13] and impaired renal elimination. As both lactate and phosphate elevation are dependent on sufficient organ perfusion, an increase in both parameters may forecast a higher risk of mortality, which is illustrated by a moderate relationship in our correlation analyses (Figure 2D and Figure 5C). In contrast, as their elimination is distinct (lactate is eliminated primarily by the liver [32], but phosphate by the kidney [8]), and their possibly different kinetics following CA are currently open to question, phosphate may contribute to precise prognostication not compared but supplementary to lactate.

We tested the predictive role of phosphate on in-hospital survival by analyzing a mixed collective of OHCA and IHCA patients who achieved ROSC during CPR from a single center. To date, the existing literature about the CA forms has shown wildly divergent results. For example, while some studies showed a difference between the CA forms [3], others found a similar survival between the CA groups [34]. In addition, there were frequently significant differences in ROSC rates for the two forms. However, the demographic characteristics of patients in the two CA forms were often similar [3]. To better understand this prognostic value in our collective, we stratified between the two subgroups of patients from the University Clinic of Duesseldorf. In patients with IHCA, the prognostic value of the initial phosphate levels was consistent with the results from the entire collective. The AUC in ROC analyses was similar, and elevations >2.7 mmol/L predicted correctly a significant higher probability of mortality within the first 30 days post-CPR (Figure 4A). When surviving IHCA patients were subdivided according to their neurological status at discharge, those with a better status initially also had lower phosphate levels (Figure 4B). Several of the findings from the entire collective were also reflected in the analyses of the OHCA group. Elevated phosphate values predicted a higher risk of mortality, with a moderate but significant discrimination in the ROC, and OHCA patients with initial levels >2.7 mmol/L presented higher 30-day mortality (Figure 4C). In contrast to IHCA patients, surviving OHCA patients with good/moderate neurological development showed only a trend toward lower initial phosphate levels (Figure 4D), which may be explained by a small number of analyzed patients, as only 28 of the initial 159 OHCA survived to hospital discharge.

Taken together, our results strength the independent relationship between initial serum phosphate levels and the risk of mortality and neurological impairment in CA patients following ROSC. Phosphate elevations, especially >2.7 mmol/L, predict a lower chance of survival.

## 5. Limitations

Our study has several limitations. First, as collectives of patients were analyzed retrospectively, no causal connections between initial phosphate levels and time-delayed outcome were possible. Second, the cut-off value to predict a higher risk of mortality was concluded from a single-center cohort. We were able to partially circumvent this limitation by validating our results in a larger, multicenter patient collective (eICU database). In the multiple logistic regression model, we did not add further variables, which might have influenced the OR of the initial phosphate levels. In the eICU database, no data on ROSC were available, so the analysis had to be performed on all patients. Interestingly, our cut-off value discriminated a high risk of mortality also in the entire collective from the eICU database (including the ones which did not achieve ROSC), raising the question of whether the prediction of mortality could be applied to all patients. Lastly, although we adjusted the initial creatinine level after hospitalization in the OR of phosphate, acute (to chronic) kidney injury, as they might have been induced by CPR, could have led to acute elevations in phosphate levels. Future studies should examine phosphate levels post-CPR, taking into account the dynamics of renal function before and after CA to improve the adjustment and overall predictive value of phosphate. 

## 6. Conclusions

Initial serum phosphate levels >2.7 mmol/L predict a higher chance of mortality after CA following ROSC. Elevations of phosphate levels after CA following ROSC forecast a poor neurological outcome in surviving CA patients. Diagnostic algorithms should include initial phosphate assessment in CA patients as they can make individuals’ prognostication more precise.

## Figures and Tables

**Table 1:** Demographics of the CA patients treated at the University Clinic of Düsseldorf.

Parameter	Value (%)
*N*	488
Age, years	69.88 ± 12.99
Male, *n* [%]	304 [62.3%]
**CPR Details**	
IHCA	329 [67.4%]
CPR duration (min) ^a^	26.7 ± 26.7
Arrest witnessed (%) ^b^	357 [83.0%]
Shivering induction	184 [37.7%]
**Cause of arrest (%)**	
Cardiogenic shock (NSTEMI, STEMI)	207 [42.4%]
Respiratory/Hypoxia/LAE	87 [17.8%]
Shock (excl. Cardiogenic shock)	36 [7.4%]
Bleeding/Aortic dissection	20 [4.1%]
Metabolic disorder/Electrolyte	14 [2.9%]
Neurological	6 [1.2%]
Unknown	118 [24.2%]
**Cranial imaging**	213 [43.7%]
Cranial injury	43 [20.2%]
**Laboratory parameters, at index (±SD)**	
Phosphate, [mmol/L]	2.28 ± 1.1
Sodium, [mmol/L] ^c^	142 ± 8.4
Potassium, [mmol/L] ^d^	4.67 ± 1.2
Creatinine, [mg/dL] ^e^	1.88 ± 1.5
Estimated Glomerular Filtration Rate [mL/min] ^f^	48.65 ± 27.3
Hemoglobin, [g/dL] ^g^	10.6 ± 2.9
LDH (mg/dL) ^h^	1155 ± 2386
CRP [mg/dL] ^i^	5.48 ± 8
CK [U/L] ^j^	1135 ± 3019
GPT, [mg/dL] ^k^	346.6 ± 849
GOT, [mg/dL] ^l^	639 ± 1336

Missing values: ^a^ 53, ^b^ 58, ^c^ 8, ^d^ 8, ^e^ 6, ^f^ 8, ^g^ 6, ^h^ 6, ^i^ 8, ^j^ 8, ^k^ 7, ^l^ 14.

**Table 2:** Demographics of the collective from the eICU database.

Parameter	Value (%)
*N*	3299
Age, years ^a^	65.8 ± 15.5
Intrahospital mortality	1115 [34.5%]
**Gender**	
Male, *n* [%]	1872 [56.7%]
Female, *n* [%]	1424 [43.2%]
Unknown	3 [0.0%]
**Race (%)**	
Caucasian	2498 [75.7%]
African American	407 [12.3%]
Hispanic	139 [4.2%]
Asian	41 [1.2%]
Native American	24 [0.7%]
Other/Unknown	190 [5.8%]
**Laboratory parameters, maximum value on the first day (±SD)**	
Phosphate, [mg/dL]	4.4 ± 2.3
Lactate, [mg/dL] ^b^	5.2 ± 4.9
Sodium, [mmol/L] ^c^	140 ± 5.4
Potassium, [mmol/L] ^d^	4.5 ± 0.9
Creatinine, [mg/dL] ^e^	2.07 ± 2.0

^a^ for patients older than 89 years, the age was set to 89 years; missing values: ^b^ 1599, ^c^ 28, ^d^ 23, ^e^ 28.

## Data Availability

The data used for the current study can be made available by the corresponding author on reasonable request.

## References

[1] Yan S., Gan Y., Jiang N., Wang R., Chen Y., Luo Z., Zong Q., Chen S., Lv C. (2020). The global survival rate among adult out-of-hospital cardiac arrest patients who received cardiopulmonary resuscitation: A systematic review and meta-analysis. Crit. Care.

[2] Alao D.O., Mohammed N.A., Hukan Y.O., Al Neyadi M., Jummani Z., Dababneh E.H., Cevik A.A. (2022). The epidemiology and outcomes of adult in-hospital cardiac arrest in a high-income developing country. Resusc. Plus.

[3] Høybye M., Stankovic N., Holmberg M., Christensen H.C., Granfeldt A., Andersen L.W. (2021). In-Hospital vs. Out-of-Hospital Cardiac Arrest: Patient Characteristics and Survival. Resuscitation.

[4] Karam N., Marijon E., Dumas F., Offredo L., Beganton F., Bougouin W., Jost D., Lamhaut L., Empana J.-P., Cariou A. (2017). Characteristics and outcomes of out-of-hospital sudden cardiac arrest according to the time of occurrence. Resuscitation.

[5] Sandroni C., Nolan J., Cavallaro F., Antonelli M. (2007). In-hospital cardiac arrest: Incidence, prognosis and possible measures to improve survival. Intensive Care Med..

[6] Kritmetapak K., Kumar R. (2021). Phosphate as a Signaling Molecule. Calcif. Tissue Int..

[7] Berndt T., Thomas L.F., Craig T.A., Sommer S., Li X., Bergstralh E.J., Kumar R. (2007). Evidence for a signaling axis by which intestinal phosphate rapidly modulates renal phosphate reabsorption. Proc. Natl. Acad. Sci. USA.

[8] Marks J., Debnam E.S., Unwin R.J. (2010). Phosphate homeostasis and the renal-gastrointestinal axis. Am. J. Physiol. Physiol..

[9] Tonelli M., Sacks F., Pfeffer M., Gao Z., Curhan G. (2005). Relation Between Serum Phosphate Level and Cardiovascular Event Rate in People With Coronary Disease. Circulation.

[10] Tsai T.-Y., Hsu P.-F., Wu C.-H., Yang Y.-L., Chen S.-C., Huang S.-S., Chan W.L., Lin S.-J., Chen J.-W., Pan J.-P. (2021). Association between phosphate and long-term outcome in CAD patients underwent coronary intervention. Sci. Rep..

[11] Ess M., Heitmair-Wietzorrek K., Frick M., Umlauf N., Ulmer H., Poelzl G. (2013). Serum phosphate and long-term outcome among patients with stable heart failure. J. Card. Fail..

[12] Bleske B.E., Song J., Chow M.S.S., Kluger J., White C.M. (2001). Hematologic and Chemical Changes Observed during and after Cardiac Arrest in a Canine Model—A Pilot Study. Pharmacother. J. Hum. Pharmacol. Drug Ther..

[13] Makino J., Uchino S., Morimatsu H., Bellomo R. (2005). A quantitative analysis of the acidosis of cardiac arrest: A prospective observational study. Crit. Care.

[14] Jung Y.H., Lee B.K., Jeung K.W., Youn C.S., Lee D.H., Lee S.M., Heo T., Min Y.I. (2018). Prognostic value of serum phosphate level in adult patients resuscitated from cardiac arrest. Resuscitation.

[15] Bae D.H., Lee H.Y., Jung Y.H., Jeung K.W., Lee B.K., Youn C.S., Kang B.S., Heo T., Min Y.I. (2021). PROLOGUE (PROgnostication using LOGistic regression model for Unselected adult cardiac arrest patients in the Early stages): Development and validation of a scoring system for early prognostication in unselected adult cardiac arrest patients. Resuscitation.

[16] Heo W.Y., Jung Y.H., Lee H.Y., Jeung K.W., Lee B.K., Youn C.S., Choi S.P., Park K.N., Min Y.I., on behalf of the Korean Hypothermia Network Investigators (2022). External validation of cardiac arrest-specific prognostication scores developed for early prognosis estimation after out-of-hospital cardiac arrest in a Korean multicenter cohort. PLoS ONE.

[17] Luescher T., Mueller J., Isenschmid C., Kalt J., Rasiah R., Tondorf T., Gamp M., Becker C., Sutter R., Tisljar K. (2019). Neuron-specific enolase (NSE) improves clinical risk scores for prediction of neurological outcome and death in cardiac arrest patients: Results from a prospective trial. Resuscitation.

[18] Schick A., Prekker M.E., Kempainen R.R., Mulder M., Moore J., Evans D., Hall J., Rodin H., Larson J., Caraganis A. (2022). Association of hypoxic ischemic brain injury on early CT after out of hospital cardiac arrest with neurologic outcome. Am. J. Emerg. Med..

[19] Donnino M.W., Andersen L.W., Giberson T., Gaieski D., Abella B., Peberdy M.A., Rittenberger J.C., Callaway C.W., Ornato J., Clore J. (2014). Initial lactate and lactate change in post-cardiac arrest: A multicenter validation study. Crit. Care Med..

[20] Bruno R.R., Wernly B., Binneboessel S., Baldia P., Duse D.A., Erkens R., Kelm M., Mamandipoor B., Osmani V., Jung C. (2020). Failure of Lactate Clearance Predicts the Outcome of Critically Ill Septic Patients. Diagnostics.

[21] Levey A.S., Stevens L.A., Schmid C.H., Zhang Y.L., Castro A.F., Feldman H.I., Kusek J.W., Eggers P., Van Lente F., Greene T. (2009). A new equation to estimate glomerular filtration rate. Ann. Intern. Med..

[22] Haschemi J., Erkens R., Orzech R., Haurand J.M., Jung C., Kelm M., Westenfeld R., Horn P. (2021). Comparison of two strategies for managing in-hospital cardiac arrest. Sci. Rep..

[23] Hruska K.A., Mathew S., Lund R., Qiu P., Pratt R. (2008). Hyperphosphatemia of chronic kidney disease. Kidney Int..

[24] Mandrekar J.N. (2010). Receiver Operating Characteristic Curve in Diagnostic Test Assessment. J. Thorac. Oncol..

[25] Yanta J., Guyette F., Doshi A.A., Callaway C., Rittenberger J.C. (2013). Renal dysfunction is common following resuscitation from out-of-hospital cardiac arrest. Resuscitation.

[26] Miller C.J., Doepker B.A., Springer A.N., Exline M.C., Phillips G., Murphy C.V. (2018). Impact of Serum Phosphate in Mechanically Ventilated Patients With Severe Sepsis and Septic Shock. J. Intensive Care Med..

[27] Li Z., Shen T., Han Y. (2022). Effect of Serum Phosphate on the Prognosis of Septic Patients: A Retrospective Study Based on MIMIC-IV Database. Front. Med..

[28] Kim D.W., Jung W.J., Lee D.K., Lee K.J., Choi H.J. (2021). Association between the initial serum phosphate level and 30-day mortality in blunt trauma patients. J. Trauma Acute Care Surg..

[29] Chen Y., Luo M., Xu H., Zhao W., He Q. (2021). Association between serum phosphate and mortality in critically ill patients: A large retrospective cohort study. BMJ Open.

[30] Yang J., Cheng Y., Wang R., Wang B. (2022). Association between early elevated phosphate and mortality among critically ill elderly patients: A retrospective cohort study. BMC Geriatr..

[31] Rugg C., Bachler M., Kammerlander R., Niederbrunner D., Bösch J., Schmid S., Kreutziger J., Ströhle M. (2021). ICU-Admission Hyperphosphataemia Is Related to Shock and Tissue Damage, Indicating Injury Severity and Mortality in Polytrauma Patients. Diagnostics.

[32] Kraut J.A., Madias N.E. (2014). Lactic Acidosis. N. Engl. J. Med..

[33] Wadsworth R., Siddiqui S. (2016). Phosphate homeostasis in critical care. BJA Educ..

[34] Andersson A., Arctaedius I., Cronberg T., Levin H., Nielsen N., Friberg H., Lybeck A. (2022). In-hospital versus out-of-hospital cardiac arrest: Characteristics and outcomes in patients admitted to intensive care after return of spontaneous circulation. Resuscitation.

